# Key Aspects of Nucleic Acid Library Design for in Vitro Selection

**DOI:** 10.3390/ijms19020470

**Published:** 2018-02-05

**Authors:** Maria A. Vorobyeva, Anna S. Davydova, Pavel E. Vorobjev, Dmitrii V. Pyshnyi, Alya G. Venyaminova

**Affiliations:** 1 Institute of Chemical Biology and Fundamental Medicine, Siberian Division of Russian Academy of Sciences, Lavrentiev Ave., 8, 630090 Novosibirsk, Russia; anna.davydova@niboch.nsc.ru (A.S.D.); vorobyev@niboch.nsc.ru (P.E.V.); pyshnyi@niboch.nsc.ru (D.V.P.); ven@niboch.nsc.ru (A.G.V.); 2 Department of Natural Sciences, Novosibirsk State University, Pirogova St., 2, 630090 Novosibirsk, Russia

**Keywords:** SELEX, aptamers, design of nucleic acid libraries

## Abstract

Nucleic acid aptamers capable of selectively recognizing their target molecules have nowadays been established as powerful and tunable tools for biospecific applications, be it therapeutics, drug delivery systems or biosensors. It is now generally acknowledged that in vitro selection enables one to generate aptamers to almost any target of interest. However, the success of selection and the affinity of the resulting aptamers depend to a large extent on the nature and design of an initial random nucleic acid library. In this review, we summarize and discuss the most important features of the design of nucleic acid libraries for in vitro selection such as the nature of the library (DNA, RNA or modified nucleotides), the length of a randomized region and the presence of fixed sequences. We also compare and contrast different randomization strategies and consider computer methods of library design and some other aspects.

## 1. Introduction

Nucleic Acid (NA) aptamers [1] are a special class of nucleic acid molecules capable of tight and specific binding with certain molecular or supramolecular targets, thanks to characteristic spatial structures. The range of their targets is enormously wide. Nowadays, NA aptamers have been generated to metal ions (e.g., mercury [2] and lead [3]), small organic molecules (e.g., theophylline [4] and cocaine [5]), larger molecules (e.g., fluorophores [6,7] and porphyrins [8]), peptides and proteins (e.g., hormones [9,10], enzymes [11,12], antibodies [13] and cell surface proteins [14]) and liposomes [15]. These are just a few examples selected from a large diversity of NA aptamers. Nucleic acid aptamers were selected from the NA libraries by means of the method of Selective Evolution of Ligands by Exponential enrichment (SELEX) [16,17]. SELEX technology incorporates a variety of related methods for selecting functional nucleic acids with the desired properties, including also catalytic nucleic acids and riboswitches [18,19]. A selection process could also be aimed at finding genomic sequences or expressible NAs with an affinity to a specific molecule, e.g., to reveal the sequence specificity of NA-enzyme interactions [20,21]. In this review, we focus particularly on NA aptamers.

The main characteristics of NA aptamers are defined by their chemical nature. As nucleic acids, these molecules possess a significant negative charge and are susceptible to nuclease hydrolysis, and surrounding conditions (pH, ionic strength and the presence of certain ions) can influence the stability of their secondary structure. Binding with a target molecule, the aptamer can change the properties of the target, e.g., inhibit the enzymatic activity [11] or alter the characteristics of fluorescent dyes [22].

The molecular recognition function specifies the areas of possible applications of NA aptamers. An ability to inhibit pathogenic proteins affords an opportunity to employ aptamers as therapeutics [22,23,24,25]. Aptamers specific to certain cell-surface receptors, which are able to induce an internalization process, could be used as vehicles for cell-targeted drug delivery [26]. Aptamers are anticipated to compete with therapeutic monoclonal antibodies since the chemical synthesis of nucleic acids is far simpler and more cost-effective than obtaining humanized antibodies (although the SELEX process itself could become rather laborious). A set of chemical modifications is available to improve the nuclease resistance and pharmacokinetics of NA aptamers [27]. It is also worth noting that aptamers have the benefit of having a low immunogenicity typical for most oligonucleotides.

Bioanalytics represents probably the broadest application area of nucleic acid aptamers. In principle, every aptamer can be considered as a recognizing module for a certain molecule. It is no wonder that such a vast diversity of aptamer-based biosensors (also known as “aptasensors”) has been created (see [28,29,30,31] for a review).

**Figure 1:**
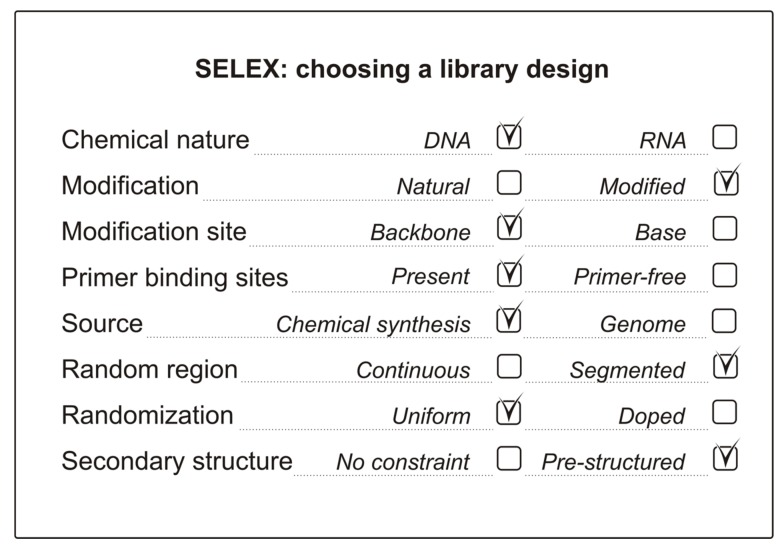
An example checklist for an NA library design with the key issues to be considered.

The main success criteria for any given aptamer include binding affinity, nuclease resistance and convenience of chemical synthesis. All these properties are largely defined by the particular nucleic acid library employed for SELEX. Therefore, the choice of library design has a great impact on the overall efficiency of the selection. When generating the initial library, a researcher should keep in mind the properties of the target (such as in capture SELEX for small molecules [32]) and the end use of an aptamer (whether nuclease resistance is necessary or not) [27,33]. The importance of covering a maximal sequence space (a multi-dimensional space of different sequences of a certain length), the necessity of introducing a particular sequence or structural element should also be taken into account. In some cases, additional effort is needed to obtain a library that enables the generation of aptamers to SELEX-inaccessible (somewhat similar to non-immunogenic) targets [34,35]. Thus, at the beginning of the study, one has to fill out a kind of checklist of the key issues to choose the most suitable library design (Figure 1). The main aspects regarding the design of the initial libraries for aptamer selection and the basic trends in library design will be reviewed and discussed below.

## 2. General Issues of Initial Library Design

### 2.1. DNA or RNA?

All SELEX studies can be generally divided into two groups. In the first group, the choice of the type of nucleic acid library is predetermined by the task of the study such as for the in vitro selection of ribozymes, riboswitches, DNAzymes or genomic SELEX studies. Experiments on the isolation of RNA aptamers or artificial riboswitches intended to be expressed in cells also relate to this group. The second group includes SELEX studies on aptamers that will be further employed for research, therapeutic or bioanalytical purposes. In this case, a researcher can deliberately choose the type of sugar-phosphate backbone.

The first decade in the development of SELEX technology was marked by a dominance of RNA aptamers [36,37]. This was possibly due to the common opinion that only RNA molecules could form functional motifs [38]. At the very beginning of the SELEX era, Ellington and Szostak demonstrated the ability of single-stranded DNA to fold into functional spatial structures [39]. Nevertheless, until 2007, about 70% of all experiments in the field related to RNA aptamers [36]. The distribution became quite the opposite in 2008–2013: DNA aptamers now occupy 70% of SELEX studies, and no significant differences were found in the distributions of the lowest *K*_D_ values [36]. DNA and RNA aptamers generated for a number of small-molecule targets have demonstrated similar affinities [40].

Thus, neither the RNA nor DNA libraries provide any systemic preferences for the isolation of affine aptamers [36]. Such preferences can clearly be attributed to some modified nucleic acids, e.g., Slow Off-rate Modified Aptamers (SOMAmers), which will be discussed below. The particular conditions of an aptamer’s application also influence the choice of a sugar-phosphate backbone. An enhanced nuclease resistance could require the use of backbone chemical modifications, which will be briefly described in the next section. According to [37], the number of aptamers isolated from non-natural nucleic acid libraries increased significantly in 2011–2015.

### 2.2. Backbone Modifications of NA Libraries

**Figure 2:**
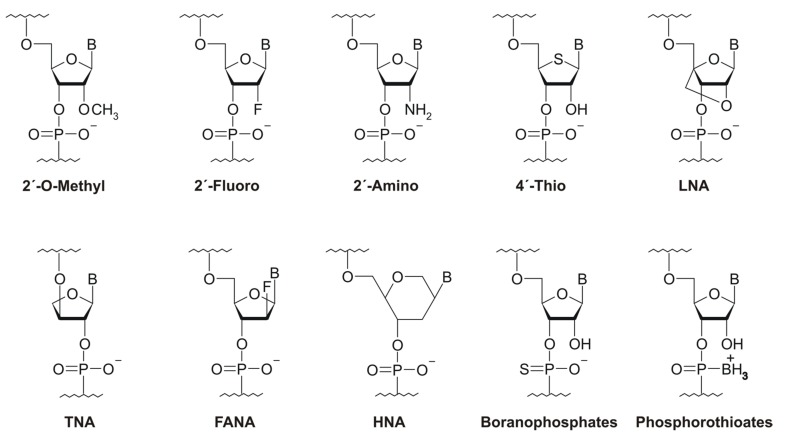
Sugar-phosphate backbone modifications compatible with a SELEX procedure. LNA: locked nucleic acids; TNA: threose nucleic acid; FANA: fluoroarabino nucleic acid; and HNA: 1,5-anhydro hexitol nucleic acid.

A number of popular applications of in vitro selected aptamers—such as the design of new therapeutics or engineering of drug delivery systems and biosensors—assumes their use in biological media containing different nucleases. Both DNA and RNA aptamers are susceptible to nuclease degradation. To protect them, a large set of chemical modifications of the sugar-phosphate backbone has been developed. However, any post-selective chemical modification of individual aptamers can affect binding affinity, so the modification pattern should be optimized in every particular case, which is rather laborious and time-consuming. Therefore, it seems reasonable to introduce modified nucleotides into the initial library to select molecules that are both affine and nuclease-resistant. One of the most important criteria for such pre-SELEX modifications is the compatibility of modified nucleotides with all enzyme reactions involved in a selection protocol. A number of chemical modifications meeting this requirement are now available (see the reviews in [27,35,41,42,43]), including ribose (2′-NH_2_, 2′-F, 2′-O-Me, 4′-S-, LNA (locked nucleic acids), TNA (threose nucleic acid), FANA (fluoroarabino nucleic acid) and HNA (1,5-anhydro hexitol nucleic acid)) and internucleoside phosphate (boranophosphate or phosphorothioate) modifications (Figure 2). Among them, 2′-modifications are clearly at the top of the list. The first SELEX-compatible 2′-modification was the replacement of ribose 2′-OH by an amino group [44]. However, this type of modification was then quite rarely used, owing to problems with the chemical synthesis of 2′-NH_2_-modified aptamers and the negative impact of the 2′-amino group on the ribose conformation [35]. In contrast, the 2′-F modification of pyrimidine nucleotides, which was proposed almost at the same time, gained outstanding popularity since it provided sufficient nuclease resistance, did not dramatically affect the RNA spatial structure and could be introduced by even using a non-modified T7 RNA polymerase under optimized conditions [45]. To apply any other SELEX-compatible modifications as mentioned above, one should use mutant versions of polymerase enzymes (see [41,46,47] for reviews).

### 2.3. The Length of the Random Region

When choosing the length of the random region, a researcher should consider both the sequence space and structural diversity. In the general case, the maximum possible sequence space for a random sequence of N nucleotides comprises a total of 4^N^ possible sequences. Therefore, for those quantities of libraries that can be routinely obtained and handled, a maximal theoretical diversity can only be reached for random regions shorter than 28 nt (7 × 10^16^ sequences ≈ 0.1 µmol corresponds to a fully-represented library) [48]. Longer libraries are unable to extensively cover the sequence space. On the other hand, longer sequences can fold into more complex structures that may be needed to form a target-binding domain. Thus, a balance should be kept between the diversity of the sequences and the desired complexity of the spatial structures formed by these sequences. For in vitro selection of aptamers, 30–50-nt randomized regions are the most abundant [49].

With regard to the minimal sequence diversity to provide a sufficient selection, a value of 10^11^ is often used (see [50]), based on SELEX publications from the early 1990s [1,51,52]. It should be noted that all these works deal with RNA SELEX to small-molecule targets, so the question arises as to whether such estimation is applicable for all possible types of targets and libraries.

Aside from the theoretical considerations, from a practical point of view, the length of the library is governed by: (1) the convenience and cost of its chemical synthesis; (2) the possibility of PCR (polymerase chain reaction) artifact formation in the course of an amplification of long libraries; and (3) future applications of the selected aptamers. When an aptamer is further used for practical applications, a shorter length of the oligonucleotide chain is always better. To minimize the length of an individual aptamer, a series of its truncated variants has to be synthesized and tested to choose the minimal one retaining target binding affinity. To avoid this resource-consuming procedure, Thiel et al. [50] employed a short 51-nt library with a randomized region as short as 20 nt and demonstrated that this length was sufficient to generate high-affinity 2′-F-RNA aptamers to protein targets.

### 2.4. Primer-Binding Sites and Primer-Free SELEX

**Figure 3:**
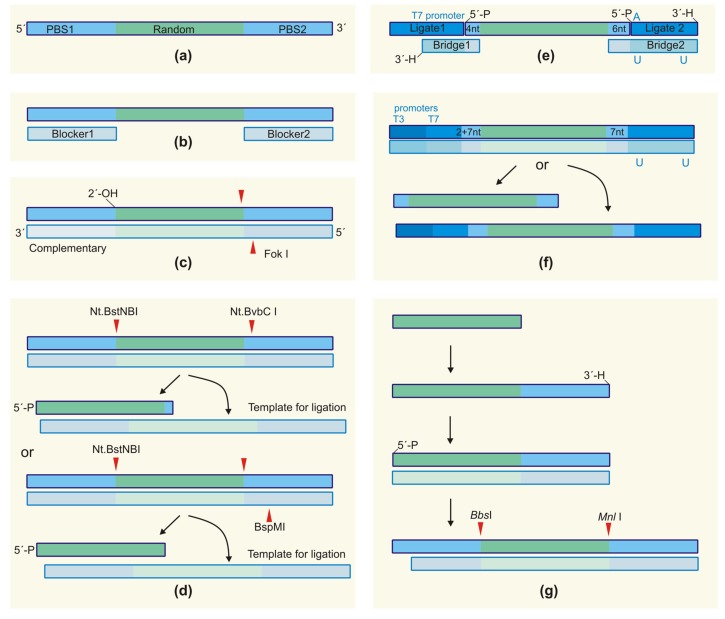
Different variants of design for NA libraries for a primer-free SELEX. (**a**) A conventional NA library; (**b**) blocked primer-binding sites for primer-annealing SELEX [62]; (**c**) the design of primer-binding sites for primer-free genomic SELEX [64]; (**d**) the design of a DNA library for primer-free SELEX from a completely randomized library [65]; (**e**) the RNA library for a tailored SELEX in a complex with auxiliary oligonucleotides [68]; (**f**) the DNA template for a dual-RNA library suitable for both conventional and tailored SELEX [69]; (**g**) DNA libraries lacking any constant nucleotides for the primer-free SELEX protocol of Lai et al. [72]. PBS: primer binding site, 2′-OH-ribonucleotide, 3′-H-dideoxynucleotide.

Traditional SELEX protocols, which are still prevalent today, imply the use of two fixed sequences flanking the randomized region for primer annealing during amplification (Figure 3a). As a rule, primer-binding sites (PBS) are about 20 nt in length. According to the statistical analysis performed in [49], their length does not correlate with the length of a randomized region. The sequences of primer-binding sites are designed to meet several general requirements, particularly to avoid PCR artifacts emerging from self-association or secondary structure formation and to ensure efficient polymerase extension. In the case of RNA SELEX, the 5′-primer contains a promoter sequence for T7 RNA polymerase. A detailed guide to the design of the primer-binding sites can be found in [53]. Some examples of starting SELEX libraries and primers are given in the Table 1.

Ideally, aptamer sequences generated by in vitro selection should bind their targets by means of spatial structures formed only by nucleotides from a random region. For most aptamers, this is indeed the case: the analysis of >2000 sequences from the Aptamer Database revealed that for a majority of aptamers, their secondary structure was independent of primer-binding sites [49]. However, there was a number of outliers (examples in [59,60,61]). Taking this into account, primer-binding sites cannot be simply cut off to minimize the length of the sequence during aptamer truncation, and additional minimization studies are needed. Moreover, during the SELEX, primer-binding sites could interact with sequences in the random region, hampering their target binding and/or amplification (for more details, see [62] and the references therein). 

These problems stimulated a search for SELEX approaches that minimize the influence of primer-binding sites on the sequence and structure of selected aptamers (schematically depicted in Figure 3). For instance, Shtatland et al. [61] showed that fixed regions of a genomic RNA library (with *Escherichia coli* (*E. coli*) genome fragments as a random region) interacted with a random region, which resulted in a large number of experimental artifacts. After the traditional SELEX from this library on MS2 bacteriophage capsid protein, about 90% of the generated sequences were represented by artifacts (not found in the *E. coli* genome). The authors proposed two alternative selection strategies to neutralize the negative impact of constant regions: primer-annealing genomic SELEX and primer-switching genomic SELEX. In the primer-annealing genomic SELEX protocol, prior to selection, the RNA library was hybridized with two oligonucleotides complementary to the primer-binding sites (Figure 3b). This approach provided 60% of the artifacts in the obtained clones. During the course of primer-switching genomic SELEX, several rounds of classical SELEX were performed, followed by a replacement of primer-binding sites and subsequent classical or primer-annealing SELEX. To replace the flanking regions, the purified library was digested by the FokI restrictase (restriction sites were introduced 9–13 nt from the random region); the sticky ends were extended to blunt ends by a Klenow reaction; then, new primer-binding sites were ligated to the library. This approach enabled the authors to decrease the fraction of unwanted products down to 10%.

Ouellet et al. successfully adapted the primer-annealing SELEX protocol for completely random libraries [62,63]. Blocking oligonucleotides annealed with primer-binding sites eliminated their negative impact in several selections on therapeutically-important targets.

The approach proposed by Shtatland et al. was further developed for the genomic SELEX on the bacteriophage Ff gene 5 protein [64]. The authors hypothesized that constant nucleotides remaining in the library after an enzymatic digestion could also influence the course of selection. In their version of primer-free genomic SELEX, the Fok1 restriction site at the 5′-end was combined with a ribose linkage at the 3′-end of the library (Figure 3c). Enzymatic digestion followed by alkaline treatment provided a genomic insert free of any constant nucleotides. To regenerate the primer-binding sites for amplification at every SELEX round, the authors employed thermal cycles of hybridization-extension using the initial genomic library as a template.

Pan et al. [65,66,67] employed the possibility of using the second strand as a template for completely randomized libraries. The authors developed two similar approaches for primer-free SELEX, which allowed the use of DNA libraries with only two constant nucleotides or even without constant positions (Figure 3d). The first approach was based on the introduction of Nt.BbvCI and Nt.BstNBI restriction sites into the initial dsDNA library. These enzymes recognize dsDNA, but cleave only one strand. A subsequent digestion of the library resulted in the formation of 32-nt ssDNA (0 + 30 + 2), which was used for in vitro selection. The second DNA strand remained uncleaved and acted as a template for the ligation of primer-binding sites prior to amplification. The second protocol provides a completely primer-less DNA library. In this case, the authors supplied the initial DNA library with Nt.BstNBI and BspMI restriction sites. Digestion by both restrictases provided the 30-nt ssDNA library (0 + 30 + 0), while the treatment only by Nt.BstNBI gave an uncleaved second strand, which also acted as a ligation template.

The possibility of using primer-free SELEX for completely randomized RNA libraries was also shown in [68]. The authors developed a tailored SELEX approach, implying the use of primers/adapters added previously by ligation and removed within the amplification processes (Figure 3e). A randomized 40-nt region was flanked by two short constant sequences (4 and 6 nt) for annealing the adapter oligonucleotides, so the total length of the aptamers generated by this method was as low as 50 nt. Further development of the method led to the design of the dual RNA library [69]. An introduction of both T3 and T7 RNA promoters (Figure 3f) allowed the generation of two different RNA libraries. The transcription carried out by a T3 RNA polymerase provided a long “traditional” RNA library with 34-nt random regions and conventional primer binding sites. Alternatively, the use of T7 RNA polymerase obtained an RNA library for tailored SELEX, with the same N34 region flanked by two short fixed sequences forming a stem that excluded their involvement in active functional structures. The design of primer-binding sequences complementary to each other was also employed in [70]. It is noteworthy that such stem-forming flanking sequences could, in some cases, hamper the selection of aptamers [71].

Another protocol for primer-free SELEX was developed by Lai et al. [72,73] for a totally randomized 30-nt DNA library aimed at selecting aptamers for HIV RT (Figure 3g). To amplify the library after target binding, the authors proposed the use of a non-template ligation of the 3′-primer-binding fragment containing the MnlI site by the thermostable RNA ligase at 60 °C. These ligation conditions are supposed to lower the possibility of secondary structure formation and increase the efficiency of ligation as compared to the conventional T4 RNA ligase. The ligation of the 5′-primer-binding site as a duplex containing the BbsI site was performed by the T4 DNA ligase.

A drastic approach to avoid the use of primer-binding sites was recently proposed by Tsao et al. [74]. The Rotating Magnetic Field Magnetic-Assisted Rapid Aptamer Selection (RO-MARAS) method enables the one-step generation of high affinity aptamers, which relies on the sophisticated, but efficient, procedure of pool isolation. The protocol included an incubation of the starting library, free of any constant nucleotides, with a target protein immobilized on the surface of magnetic beads. This was followed by the employment of a rotating magnetic field to select the most tightly bound molecules. Notably, the amplification of the enriched library before sequencing required a very complex scheme to add primer-binding sites.

To summarize, a number of different initial libraries and selection schemes are now available to generate the aptamers lacking primer-binding sites. We would like to emphasize that the absence of fixed flanking sequences provides the important advantages of (1) decreasing the probability of SELEX artifacts; and (2) shortening the overall length of the aptamer sequence. At the same time, all primer-free SELEX protocols rely on the additional stages of ligation and restrictase digestion. Insufficient ligation, or deletion of restriction sites during PCR amplification could result in a loss of some potential binders, which can be considered as the pitfall of primer-free selection.

### 2.5. NA Libraries Containing Additional Constant Sequences

**Figure 4:**
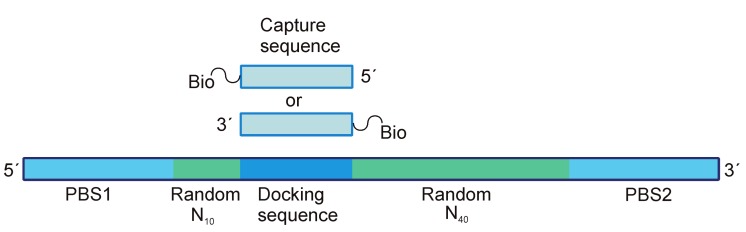
A general scheme of library design for a capture SELEX.

It should be mentioned that primer-binding sites are not the only possible constant regions of the library having an auxiliary role. NA libraries can also be supplied by additional constant sequences necessary for the immobilization within a capture SELEX approach. This approach, first proposed by Nutiu et al. [75,76] for the selection of structure-switching aptamers specific to ATP (adenosine triphosphate) or GTP (guanosine triphosphate), relies on the annealing of the so-called docking sequence within a library to the complementary capture oligonucleotide bound to a carrier through biotin–streptavidin interactions (Figure 4). In this way, prior to selection, the initial library is immobilized on a carrier, and target binding causes a structural rearrangement, which results in duplex dissociation and passing of the library to the solution. Therefore, the pool without target binding affinity remains immobilized and can be easily separated from the enriched one. Aptamers selected by this method gain the ability of structure-switching, which can be employed for engineering analytical systems (e.g., fluorescent beacons) for the detection of target molecules. A capture SELEX method turned out to be particularly suitable for selecting aptamers on small-molecule targets such as antibiotics, toxins, drugs or food contaminants (see the reviews in [32,77]). The problem of separating bound and unbound pools becomes crucial for these selections. After target binding, a change of the properties of NA molecules is not significant enough to isolate the complexes from unbound molecules in solution. Otherwise, the immobilization of small molecule targets masks potential binding sites and also increases the probability of selecting aptamers with an affinity to the target-carrier conjugate, but not to the target itself. Capture SELEX enables the selection of the target in its native state in solution, while employing the advantages of resin-based isolation. Some shortcomings of the method are connected with subsequent applications of structure-switching aptamers: during their binding with a target in solution, a rearrangement of the structure could be different from that of immobilized aptamers, which can influence binding affinity [32].

The design of a docking sequence for capture SELEX, namely the length and nucleotide composition, should provide both strong immobilization before target binding and sufficient dissociation afterwards [57]. As a rule, it is a heterosequence of 12–18 deoxynucleotides (see, e.g., Table 1) placed within the random region (as in [57,78,79]), or extending one of the primer-binding sites (as described in [80,81,82,83]). Currently, the capture SELEX strategy is generally employed for DNA selection, but also suits RNA libraries. For example, Morse et al. [84] isolated RNA beacon aptamers specific to tobramycin; interestingly, in this case, only a 6-nt capture deoxy oligomer was used for immobilization of the library.

### 2.6. NA Libraries for a Genomic SELEX

Genomic SELEX is employed to screen sequences within a certain genome for aptamers or regulator sequences, which interact with proteins or other ligands [85], such as DNA sequences recognized by transcription factors [86], or RNA sites bound by splicing factors [87]. Initial libraries consist of genomic DNA fragments, and the motifs obtained by this method are called “genomic aptamers” [88].

Genomic SELEX libraries are derived from the genomic DNA of a given organism by means of random priming and transcription. This allows the representation of all possible genomic aptamers within a library. The first strand of a genomic DNA library is usually synthesized by the Klenow fragment in the presence of the random primer supplied by a fixed sequence at the 5′-end. After the reaction, the excess primer is thoroughly removed. The second strand is synthesized by the same method. As a result, a set of genomic sequences is obtained, flanked by constant regions. At this step, fragments of a certain length can be isolated, e.g., by electrophoretic separation. If RNA transcription is required, the T7 promotor sequence is introduced by means of PCR with the corresponding primers.

The benefits of the genomic SELEX approach over the conventional one include the use of much more restricted sequence space and the increased probability of selecting a biologically-relevant aptamer. Since the initial library is obtained from genomic DNA, RNA selection can be performed regardless of the expression level, thus making it possible to isolate RNA motifs with a low expression level, or those expressible only at certain stages of a cell cycle. Unfortunately, non-expressible RNAs can also be obtained [85].

## 3. The Design of Initial NA Libraries for More Affine Aptamers

One of the most important issues in the design of nucleic acids libraries is the maximal selection efficiency, i.e., the highest probability of selecting tight-binding aptamers. In contrast to proteins, nucleic acids possess a very limited repertoire of functional groups. Consequently, high binding affinity is reached by combining the diversity of spatial structures with the available functional groups. Otherwise, a toolkit of functionalities can be artificially expanded by adding extra chemical modifications. Below, we discuss both of these possibilities.

### 3.1. Expanding the Chemical Repertoire of NA Libraries

A more obvious (but definitely not simpler) way to generate higher-affinity aptamers is to use additional functional groups, thereby making nucleic acid aptamers more similar to proteins.

Expanding the chemical repertoire of NA libraries enables a selection of either better binders or aptamers directed to target epitopes inaccessible for unmodified pools. Additional chemical functions are generally introduced into heterocyclic bases (thoroughly reviewed in [35]).

SomaLogic, one of the world’s leading companies in the development of aptamers, has created so-called SOMAmers, or Slow Off-rate Modified Aptamers. SOMAmers are selected from base-modified nucleic acids libraries [56,89,90,91,92,93] (see Table 1 for example sequences of the library and primers). Heterocyclic base modifications introduce protein-like functionalities, which provide a unique aptamer-target complex stability and even make it possible to select aptamers for previously inaccessible targets. Novel hydrophobic base modifications for DNA libraries have also been recently proposed by Chudinov et al. [94].

**Figure 5:**
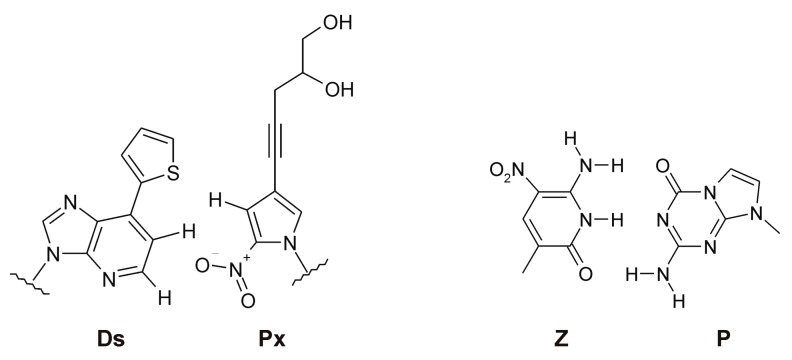
Chemical structures of artificial base pairs Ds:Px [95] and Z:P [96].

Heterocyclic base modification can also expand the genetic alphabet of nucleic acid libraries. The use of an extra artificial base pair Ds:Px (Figure 5) in the starting library was proposed by Kimoto et al. [95] to select VEGF-165 (vascular endothelial growth factor) binding aptamers. The selected aptamers, which contained several artificial base pairs, possessed 100-fold higher binding affinity as compared to the non-modified analogs. Sefah et al. [96] supplemented four natural bases with non-natural nucleosides Z and P (Figure 5) to generate DNA aptamers binding to liver cancer cells with nanomolar affinities. 

Click-SELEX represents a relatively new method for introducing chemical modifications into NA libraries. In this case, thymidine residues within a DNA library are replaced by C5-ethynyl-2′-deoxyuridine, followed by the Cu(I)-catalyzed cycloaddition of the azide component. The modified library is then employed in the modified SELMA (SELection with Modified Aptamers) protocol for different targets [97,98,99,100,101,102]. For example, this method was used to generate glycan-conjugated aptamers. Interestingly, in this case, the DNA aptamer served as a scaffold to provide an optimal tertiary structure and flexibility for the glycoclusters, which were then used as vaccine components. 

Notably, expanding the chemical repertoire of NA libraries requires base-modified nucleotide monomers and mutant polymerases, as well as more complex SELEX protocols. That is probably why such a promising strategy has not yet become routine.

### 3.2. Structural Repertoire of Nucleic Acid Libraries

#### 3.2.1. Uniformly Randomized Libraries

According to a widely-held point of view, all four nucleotides have to be uniformly represented in the random region of the library. An equal distribution is considered to provide the maximal sequence diversity, thus increasing the probability of selecting highly affine aptamers [103,104].

Currently, protocols for chemical synthesis have been developed to provide equal nucleotide distribution in the random region, which consider the different reactivities of corresponding phosphoramidites (see [53]). Methods of high-throughput sequencing and specially-developed program packages enable the estimation of the smoothness of the randomization in terms of nucleotides or short sequences, e.g., hexanucleotides [103,105]. In the latter case, a Gaussian profile is characteristic for the balanced library.

Unfortunately, today, only a few studies devoted to the impact of nucleotide composition on the structure of the library have been published. For example, the computer analysis of the structure distribution for random regions of RNA libraries revealed that for the 40-nt region, a shift to G and C (30% each) led to the predominant formation of structures with more stems when compared to the same A + U shift [106]. At the same time, for the 100-nt random region, such bias in nucleotide composition was not significant and did not markedly change the distribution of secondary structures.

On the other hand, several experiments on RNA SELEX from smoothly-randomized starting libraries have shown that the selection progress is accompanied by an accumulation of pyrimidine-rich sequences and the loss of adenosine [50,104], both for targeted and non-targeted selections. The loss of adenosine was observed for all adenosine-containing dinucleoside pairs. This corresponded to a decrease in the overall minimum free energy of the RNA library, which resulted in RNA sequences with higher predicted structural stability [50]. Therefore, a slight bias in the initial library, especially a pyrimidine bias, can be considered as acceptable, since over the course of selection, the nucleotide distribution will inevitably shift.

#### 3.2.2. Doped and Segmented NA Libraries

When a starting library is designed to improve the properties of existing aptamers by determining their target binding sites or for a functional analysis of natural RNA, the task is not a total randomization, but a delicate varying of particular nucleotides within a certain sequence. To solve this problem, one should choose doped or segmented NA libraries.

In their pioneering work, Bartel et al. [107] generated a doped library on the basis of the viral RNA element of the Rev protein of human immunodeficiency virus 1 (HIV-1) to identify the binding site for the protein. The 66-nt fragment of Rev-responsive element (RRE) was generated in such a way that point mutations were introduced uniformly throughout the sequence at a rate of 30% with 5% deletions (which meant that every position contained 65% of a wild-type nucleotide, 10% of each other nucleotide and 5% deletions). An example of the use of doping strategy to explore the secondary structure of the aptamer and determine its conservative positions is given in [108]. The authors doped the sequence of the aptamer specific to the ricin A-chain (generated by the conventional SELEX) at a 15% mutation rate. The doping strategy also helps to improve the affinity of the aptamer. Burke et al. [106,109] employed it for a secondary SELEX of pseudoknot aptamers for an HIV reverse transcriptase: truncated aptamer motifs found by the primary SELEX were doped at a 30% mutation rate (70% of the wild-type base and 10% of each of the other bases).

Nevertheless, how can we choose the mutation rate suitable for a particular task and sequence? To answer this question, Knight et al. [110] performed a comprehensive theoretical analysis of doped selections and developed an algorithm to select the length of the doped sequence and mutation rate depending on a given task. To search for sequences close to the wild-type, the authors recommended a low mutation rate (about 5%). If the structure space had to be extended, the mutation rate increased up to 30–50%. The concrete values for the doping scheme could be calculated by the developed method.

Apart from the doping of certain positions, segmental randomization is employed to specify the sequence or optimize the structure of an aptamer. For this, certain parts of the sequence are replaced by randomized stretches of the appropriate length. In principle, the segmental randomization can be considered as a special case of a doped randomization with a mutation rate of 75%. Usually, segments represent rather short sequences placed within certain elements of the secondary structure or other wild-type context [53]. A contrary example is given in [60], where core RNA aptamer sequences were flanked by 40- and 45-nt random regions to improve the aptamer analogs of green fluorescent protein. Longer segments provide larger structural diversity, which increases the probability of generating a better binder.

#### 3.2.3. Nonhomologous Recombination as an Alternative to the Doping Strategy

**Figure 6:**
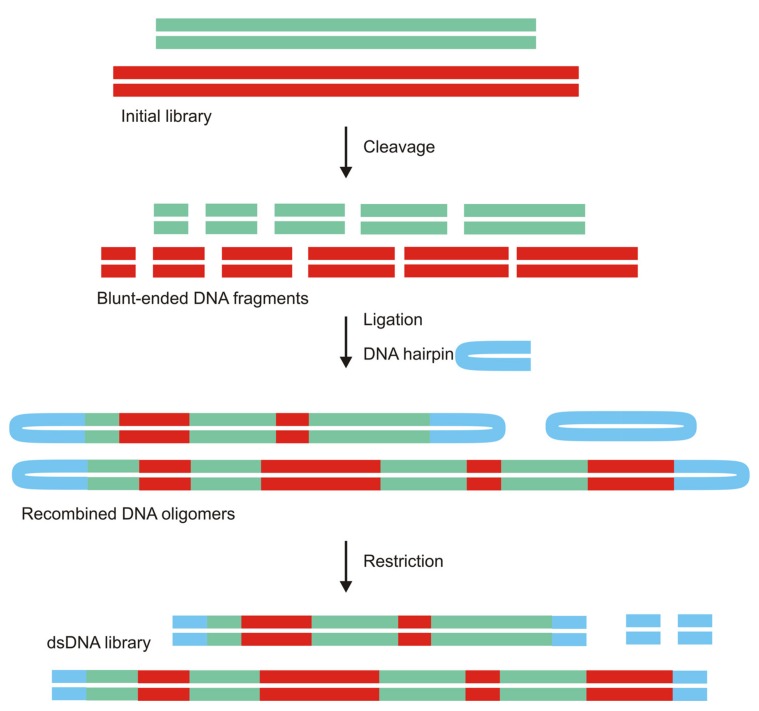
A scheme of the nonhomologous random recombination method [111].

Bittker et al. [111] proposed an entirely different approach of varying the existing aptamer sequences to find conservative regions, identify binding sites or improve the affinity: a nonhomologous random recombination (NRR). This method enables variation of the length of the library, deletion of inactive fragments and alternation of the mutual location of different motifs. For this purpose, a sequential scheme of enzymatic synthesis of NRR libraries was developed (Figure 6), starting from the treatment of the dsDNA library by DNase I and T4 DNA polymerase, which gives a mixture of blunt-ended DNA fragments. During the recombination step, DNA fragments were treated with the T4 DNA ligase under conditions favoring intermolecular ligation. The presence of an additional 5′-phosphorylated hairpin DNA containing a restriction site enabled both introducing the fixed PBS to the ends of the library and regulating the length of the recombined molecules (by varying the stoichiometry of the hairpin). Digestion of the resulted circular DNAs gave a pool of dsDNA molecules with defined sequences at both ends.

When the NRR approach had been applied to a model partly-enriched aptamer library, the authors observed that NRR-derived aptamers accumulated several copies of the active motif. Therefore, the NRR strategy was considered as a more effective alternative for error-prone PCR or site-directed mutagenesis. This strategy might also be used instead of a synthesis of doped libraries. Although the NRR protocol seems to be more complex, the synthesis of the NRR library, otherwise, does not require a sophisticated doping scheme for chemical synthesis and enables almost unlimited exploration of the sequence space. We presume that the NRR strategy could also bring benefits when used as a basic SELEX protocol starting from an unselected random pool.

#### 3.2.4. Nucleic Acid Libraries with Pre-Defined Secondary Structures

The design of starting libraries can also be performed in the framework of a paradigm that does not follow uniform randomization. An alternative concept arises from the facts that the number of productive structures providing the selection of effective binders is limited and the maximal accessible diversity of sequences folds in a restricted set of spatial structures (see [48] for a review). A computer analysis of uniformly-randomized libraries of different lengths (20–100 nt) [106] revealed that a limited set of secondary structures corresponded to every library. It was found that the complexity of the structures increased with the length of the library, and every length was characterized by three predominating structural motifs.

**Figure 7:**
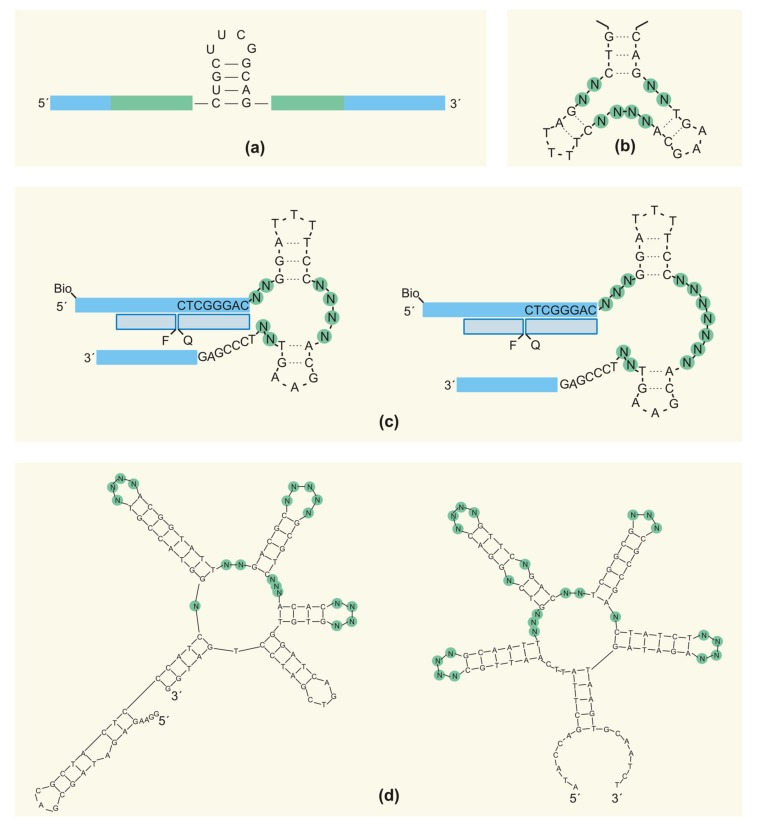
Partially-structured NA libraries. (**a**) The pre-structured RNA library with the stem-loop scaffold engineered in [58]; (**b**) the DNA library with three-way junction scaffolds for a steroid binding engineered in [115]; (**c**) DNA libraries with three-way junction scaffolds for a selection of steroid-binding DNA beacon aptamers [116]; and (**d**) highly structured RNA libraries engineered by the random filtering (left) and genetic filtering (right) approaches [123].

Thus, instead of a “smooth” randomization, it could be more beneficial to introduce secondary structure motifs into an initial library. A pioneering work in the field was published by Davis and Szostak [58]. Integrating structural data for aptamers that had been known at the time, the authors observed a common element for all structures: a stem-loop, which appeared to act as a structural anchor for recognition loops. Based on this knowledge, they designed an RNA library containing an 8-nt stem-loop motif placed in the middle of the random region (Figure 7a, Table 1). An equal mix of this pre-structured library with a conventionally-randomized one was employed in the SELEX of GTP-binding aptamers. All resulting aptamers contained the hairpin insert, thus proving the efficiency of the strategy. To further establish the proof-of-principle, the authors demonstrated that more complex structures provided more active RNAs (by examples of GTP-binding aptamers and ligase ribozymes) [112]. Notably, the hairpin motif derived in [58] was then successfully employed by other researchers to generate aptamers for different small-molecule targets [113,114].

Secondary structure elements can also be successfully introduced into DNA libraries. To form a hydrophobic pocket for steroid binding, Yang et al. [115] designed a DNA library containing a three-way junction structure with a total of eight randomized positions (Figure 7b). The same motif was also used in [116] to select structure-switchable aptamer beacons for the steroid hormone dehydroisoandrosterone 3-sulfate (Figure 7c).

Attempts were also made to design DNA libraries in a manner that provided a preferential formation of G-quadruplex structures. To generate hemin-binding G-quadruplex structures, Zhu et al. [117] created DNA libraries containing 25–45% of guanosine in the random region. The selection was successful, but the authors noted that G-rich sequences were harder to amplify by PCR, which may lead to a loss of the best binders.

Ruff et al. [118] developed a general approach for the design of pre-structured DNA libraries, also using a doping strategy. A structured DNA library with 60-nt random regions contained an RY pattern (alternating purines (R) and pyrimidines (Y)) that favors stem formation. To increase the frequency and diversity of loops and other non-stem structures within the patterned library, RY sequences alternated with stretches of 3–4 random nucleotides. Moreover, every position in the RY sites was slightly doped by nucleosides of another type: every R contained 45% A and G and 5% C and T, and vice versa for Y. The authors performed competitive selections from the mix of unpatterned and patterned libraries for three different target proteins (streptavidin, VEGF and IgE). The results proved that namely a combination of RY fragments and doping provided the selection of the highest affinity aptamers.

During the last decade, several approaches to in silico optimization of starting libraries have been developed to lower the fraction of poorly-structured (and thus low-affinity) sequences. Chushak et al. [119] developed a protocol for the computer optimization of RNA libraries prior to the selection of aptamers for small molecules. The algorithm included two main steps. First, the secondary structures of all possible sequences of a given length were analyzed. Based on secondary structure data for existing aptamers, the authors derived a set of criteria that allowed selecting an affective binder. At Step 2, 3D structures were built for all sequences meeting these criteria, followed by molecular docking with a given target molecule that resulted in a minimal free energy rating. Such high-throughput virtual screening enabled them to reduce a library of 2.5 × 10^8^ sequences to 10^3^–10^4^ sequences suitable for the experimental screening and verification.

The concepts of doped and partly-structured RNA libraries complemented each other in the method developed by Kim et al. [120]. The approach included the use of a definite set of starting sequences and certain mutation rates in certain positions within a random region (mixing matrixes). To generate these two key sets of parameters, the authors employed graph theory and matrix analysis, respectively. Starting RNA pools obtained by the proposed algorithms ensured the selection of better binders when compared to the uniformly-randomized pools. The authors also developed the web server RAGPOOLS (RNA-As-Graph-Pools) for designing and analyzing structured pools for SELEX (http://rubin2.biomath.nyu.edu/home.html) [121,122]. It is worth noting that the synthesis of the initial pool according to the mixing matrix (i.e., with an individual mutation rate for every doped position) may be laborious and time-consuming.

Luo et al. [123] developed two computational methods to generate starting DNA libraries with increased structural diversity: random filtering and genetic filtering. The random filtering approach is based on the secondary structure analysis of all sequences in the library and isolating those containing five-way junctions as the most structured elements. Then, for every such sequence, a set of mutant versions is generated with all four possible nucleotides at all positions not involved in base pairing. Random filtering thus pre-enriches the starting library with highly-structured motifs, hence increasing the probability of generating better binders. The genetic filtering approach aims to create a library with a desired distribution (either uniform or not) of all secondary structure elements (one-way, two-way, three-way, four-way and five-way junction). First, all secondary structures are analyzed for a library of a given length and the primer-binding sites. The authors recommended using 24 random positions for the pool design to provide complete sequence coverage. After secondary structure analysis, the pool is assigned a fitness score that indicates its proximity to the desired distribution of the structure elements. New generations of pool designs are obtained by selecting designs from previous generations with better (i.e., smaller) fitness scores and applying mutation, copy and crossover procedures. Typically, 500–3000 generations are needed for the best pool design. Examples of starting pools developed by random filtering and genetic filtering methods are given in Figure 7d. The pool with a uniform structure distribution was tested in a wet SELEX experiment aimed at finding ATP-binding DNA aptamers. Notably, the resulting aptamers possessed five-way junction structures, and their binding affinities were close to those for previously published aptamers from a conventionally-designed library. The authors concluded that although complexity alone could not guarantee better target binding, higher complexity structures possessed the potential to yield better aptamers. They also emphasized the importance of structural diversity, and not only structural complexity in the starting pool.

To sum up this section, the use of NA libraries with pre-designed secondary structures is a very promising strategy, which has been strongly underestimated until now. The inherent ability of nucleic acids to form complex spatial structures is used here to its full extent. A pre-structured library can be designed in silico considering the properties of a given molecular target. Once generated, the pre-structured initial library is further used in a routine SELEX protocol without any additional stages, modified nucleotides or unusual polymerases. However, it may be suggested that a combination of base-functionalized monomers with a pre-defined secondary structure would provide even more efficient starting libraries.

## 4. Conclusions

Nucleic acid aptamers generated by SELEX technology have proven themselves as highly selective and high-affinity, biospecific molecules for a number of applications. Aptamers are now considered as “chemical antibodies” with the advantages of chemical synthesis, long shelf-life and the ability to be built into almost any system of interest. In principle, aptamers can be selected for nearly any molecular or supramolecular target. However, to generate an efficient aptamer for a certain target, one should choose the most suitable SELEX protocol, and the most important issue in this case is the proper choice of an initial library. The design of a library is governed by the different parameters of a particular system such as the need for nuclease resistance, hydrophobicity, the molecular weight of a target molecule, etc. A classic design of a starting library, which still remains the most popular, uses a uniformly-randomized region flanked by two fixed primer-binding sequences. These universal “traditional” libraries are suitable for any SELEX target, from small molecules to proteins. Nevertheless, a number of alternative strategies has recently been developed. Primer-binding sites can be deleted to exclude their impact on the course of selection and to shorten the resulting aptamers. Different primer-free selection strategies have proven successful for protein targets. On the contrary, to generate an aptamer for a small-molecule target, it could be better to use the capture-SELEX technique, where the library is resin-immobilized through an additional docking sequence and the target retains its native structure. The smart design of random region enables the enrichment of a library with complex spatial structures favorable for the selection of tightly-binding motifs. The shape of the random region can be adjusted to fit the structure of the given molecule (or a class of molecules), so the “smart randomization” strategy might be recommended for any target. A chemical repertoire of initial NA libraries can also be expanded to generate better binders and to obtain aptamers for previously “SELEX-inaccessible” targets.

To summarize, a large variety of different approaches for library design is now available. A conscious choice from this diversity and the development of novel approaches to design the initial NA libraries would guarantee the generation of high-affinity aptamers for any desired ligand.

## Figures and Tables

**Table 1:** Examples of starting libraries for SELEX. SOMAmers, Slow Off-rate Modified Aptamers.

Type	Starting Libraries and Primers (5′->3′)	Ref.
Classical SELEX		
DNA	Library: GGGAGACAAGAATAAACGCTCAA-N40-TTCGACAGGAGGCTCACAACAGGC 5′-primer: GGGAGACAAGAATAAACGCTCAA 3′-primer: GCCTGTTGTGAGCCTCCTGTCGAA	[45]
RNA, 2′-F-pyrimidine (Py) modified RNA, 2′-NH_2_ Py modified RNA	Library: GGGAGACAAGAAUAAACGCUCAA-N40-UUCGACAGGAGGCUCACAACAGGC ssDNA template: GCCTGTTGTGAGCCTCCTTGTCGAA-N40-TTGAGCGTTTATTCTTGTCTCCC 5′-primer: TAATACGACTCACTATAGGGAGACAAGAATAAACGCTCAA ^1^ 3′-primer: GCCTGTTGTGAGCCTCCTGTCGAA	[45]
2′-O-Me RNA	Library: GGGAGAGAGGAACGUUCUCG-N30-GGAUCGUUACGACUAGCAUCGAUG ssDNA template: CATCGATGCTAGTCGTAACGATCC-N30-CGAGAACGTTCTCTCTCCCTATAGTGAGTCGTATTA 5′-primer: TAATACGACTCACTATAGGGAGAGGAGAGAAACGTTCTCG 3′-primer: CATCGATGCTAGTCGTAACGATCC	[54]
dRmY (2′-deoxy purine ribonucleotides, 2′-O-CH_3_ Py ribonucleotides)	Library: GGGAGAGGAGAAGGUUCUAC-N30-GCGUGUCGAUCGAUCGAUCGAUG ssDNA template: CATCGATCGATCGATCGACAGCG-N30-GTAGAACGTTCTCTCCTCTCCCTATAGTGAGTCGTATTA 5′-primer: TAATACGACTCACTATAGGGAGAGGAGAGAACGTTCTAC 3′-primer: CATCGATCGATCGATCGACAGC	[55]
SOMAmers	Library: GATGTGAGTGTGTGACGAG-N40-CACAGAGAAGAAACAAGACC, random region containing 5-(*N*-benzylcarboxamide)-2′-deoxyuridine (Bn-dU) or 5-[*N*-(1-naphthylmethyl)carboxamide]-2′-deoxyuridine (Nap-dU) in place of dT 5′-primer: GATGTGAGTGTGTGACGAG 3′-primer: GGTCTTGTTTCTTCTCTGTG	[56]
Capture SELEX		
DNA	Library: ATACCAGCTTATTCAATT-N10-TGAGGCTCGATC-N40-AGATAGTAAGTGCAATCT Capture oligonucleotide: Bio-GTC-(CH_2_CH_2_O)_6_-GATCGAGCCTCA or GATCGAGCCTCA-(CH_2_CH_2_O)_6_-GTC-Bio 5′-primer: ATACCAGCTTATTCAATT 3′-primer: AGATTGCACTTACTATCT	[57]
Pre-structured libraries		
RNA	Library: GGAGGCGCCAACTGAATGAA-N26-CUGCUUCGGCAG-N26-UCCGUAACUAGUUCGCGUCAC ssDNA template: GTGACGCGACTAGTTACGGA-N26-CTGCCGAAGCAG-N26-TTCATTCAGTTGGCGCCTCCTATAGTGAGTCGTATTACAT 5′-primer: ATGTAATACGACTCACTATAGGAGGCGCCAACTGAATGAA 3′-primer: GTGACGCGACTAGTTACGGA	[58]

^1^ Hereinafter in the table, the T7 promoter sequence is underlined.

## Abbreviations

SELEX	Selective Evolution of Ligands by Exponential enrichment
NA	Nucleic Acid(s)
PBS	Primer-Binding Site(s)
PCR	Polymerase Chain Reaction
Pu, or R	Purine nucleotide
Py, or Y	Pyrimidine nucleotide
